# Salient object segmentation based on active contouring

**DOI:** 10.1371/journal.pone.0188118

**Published:** 2017-11-27

**Authors:** Xin Xia, Tao Lin, Zhi Chen, Hongyan Xu

**Affiliations:** College of Computer Science, Sichuan University, Chengdu, Sichuan, China; Institute of Automation Chinese Academy of Sciences, CHINA

## Abstract

Traditional saliency detection algorithms lack object semantic character, and the segmentation algorithms cannot highlight the saliency of the segmentation regions. In order to compensate for the defects of these two algorithms, the salient object segmentation model, which is a novel combination of two algorithms, is established in this paper. With the help of a priori knowledge of image boundary background traits, the K-means++ algorithm is used to cluster the pixels for each region; in line with the sensitivity of the human eye to color and with its attention mechanism, the joint probability distribution of the regional contrast ratio and spatial saliency is established. The selection of the salient area is based on the probabilities, for which the region boundary is taken as the initial curve, and the level-set algorithm is used to perform the salient object segmentation of the image. The curve convergence condition is established according to the confidence level for the segmented region, thus avoiding over-convergence of the segmentation curve. With this method, the salient region boundary is adjacent to the object contour, so the curve evolution time is shorter, and compared with the traditional Li algorithm, the proposed algorithm has higher segmentation evaluation scores, with the additional benefit of emphasizing the importance of the object.

## Introduction

Salient object segmentation refers to the segmentation of important and semantic objects from an image. The correctness of the segmentation determines the effectiveness of subsequent analysis and processing. It is a key step to the analysis and understanding of images. The technology can be used in the fields of object detection, image retrieval, and editing. The traditional image segmentation algorithm is based on image features (e.g., brightness, color, and edge), and the image will be divided into several *semantic concept* areas [1–3], but the contribution of each region to an understanding of the image is not indicated. The saliency detection uses regional contrast and spatial position to analyze the saliency of each region [4, 5], but each region only has one consistent color brightness and lacks semantic meaning.

The human visual system can identify salient objects in an image quickly and accurately [6]. From the perspective of low-level features of the image, an object is formed mainly by its own characteristics, such as edges and boundaries, and the saliency of the object depends primarily on relative differences in brightness and color along with its relative spatial location. Traditional image saliency detection algorithms generate a saliency chart of pixel importance probability [7, 8]. Wang, Peng [9] proposed the binary salient segmentation estimation model [10] for images, using stochastic theory. In consonance with the color sensitivity of the human eye, a regional saliency analysis model [11] has been built that is based on the statistical characteristics of image color components. Using the different responses of the eye to signals of different frequencies, a saliency detection algorithm based on signal frequency has been designed [12]. At present, most saliency detection algorithms are based on the ability of the human eye to distinguish the boundary of blurred objects by its contrast sensitivity; through contrast analysis to estimate the image pixels and the contribution of regions to the visual composition, an image saliency analysis model can be designed. Saliency analysis is divided according to the region size into local and overall saliency models [13, 14]. Fu combined the relationship between regional contrast and relative position organically, proposing a clustering-based saliency detection algorithm [15]. Traditional saliency detection algorithms only highlight the importance of regions for image analysis; the regions lack object semantics.

In order to extract objects with semantic concepts from the image, an image segmentation algorithm is often used. A segmentation algorithm based on active contouring can effectively extract the complete objects from images [1]; a priori curve of this algorithm is combined with image areas or edge information to perform a priori object segmentation. For the sake of suppressing the influence of texture and noise on the segmentation, Mumford and Shah smoothed the images and evolved the curve segmentation model [16], but the Mumford–Shah (MS) functional is not convex, making it difficult to solve. Chan and Vess established the Chan–Vess (CV) segmentation model [17] by area means; this method is better for the segmentation of cartoon images and in addition is not sensitive to noise. In order to suppress texture, Tsai, Yezzi [18] proposed a piecewise approximation of the Piece-Smooth (PS) segmentation model, which can suppress texture to a certain extent, but the computation requirements are large. Li et al. proposed the active contour segmentation algorithm [19], which is based on edges; however, the algorithm uses the local information of the image edge, and the result is more sensitive to noise. To suppress noise, the segmentation image is often treated by Gaussian smoothing. However, Gaussian smoothing fuzzes object contours, and for weak edges the segmentation effect is not ideal. Traditional segmentation algorithms can effectively segment an image into meaningful regions, but they cannot evaluate the importance of the segmented regions to visual comprehension.

The attention mechanism of the human eye can quickly and accurately extract salient semantic objects. As the traditional saliency detection algorithm lacks object semantics and the segmentation algorithm cannot highlight segmentation region saliency, in order to address the individual deficiencies of the two algorithms, in this paper, two algorithms are organically combined to create a salient object segmentation model. Firstly, the model is based on the statistical finding that 85% of image boundaries belong to the background; this is used to establish features of the background image as prior knowledge, and through the K-means++ algorithm, the clustering pixels are divided into various regions. Secondly, regional contrast and spatial saliency are analyzed and estimated according to the human eye's sensitivity to color and its attention mechanism, the regional saliency joint probability distribution is established, and the region of saliency is selected by probability analysis. Finally, taking the saliency regional boundary as the initial curve, the level-set algorithm is used to perform the image segmentation, and the convergence condition of the curve is established according to the confidence level for the segmented region, to avoid over-convergence of the segmentation curve. In this model, each region has a clear semantic meaning, and the model highlights the importance of regional borders. After providing the details of the algorithms, this paper describes an experiment comparing the proposed method with the traditional Li algorithm in terms of the suitability of the salient region boundary, the system times required, and F-measure scores.

## Detection of salient regions

Pixels in the same region of an image should have the same attributes in the feature space. This model uses the L*a*b* color model, which can describe any color in nature, as the feature space. In this space, the three components L*, a*, and b* are perpendicular to each other, where a*, and b* indicate any color and hue characteristics, respectively.

$$\{\begin{array}{c} L^{*}=116f\left(Y/Y\right)-16\\ a^{*}=500\left[f\left(X/X_{n}\right)-f\left(Y/Y_{n}\right)\right]\\ b^{*}=200\left[f\left(X/X_{n}\right)-f\left(Z/Z_{n}\right)\right] \end{array}$$ (1)

Therein, *X*,*Y*,*Z* indicate the three components of the CIEXYZ color model and have the values 95.047, 100, and 108.083, respectively, and *f*(*t*) is defined as

$$f(t)=\left\{ \begin{array}{ll} t^{\frac{1}{3}} & t>(\frac{6}{29}{)}^{3}\\ \frac{1}{3}{\left(\frac{6}{29}\right)}^{3}+\frac{4}{29} & \;\mathrm{otherwise} \end{array}\right.$$ (2)

In the L*a*b* feature space, the image pixels are clustered into a region *R*^*k*^(*k* = 1,2,…,*m*), which enables each region to meet in the feature space: the similarity of pixels in the same region is higher, and the similarity of pixels in different regions is lower.

The K-means++ clustering algorithm is used, but the initial cluster center needs to be determined in advance; different initial cluster centers may lead to completely different clustering results. To determine the initial cluster center, images from the Internet were analyzed, and 85% of image edges were found to belong to the background region. In accordance with this property, the region which has 20 pixels to the image boundary is taken as the initial background region, and the regional mean value in the feature space is taken as the initial seed point. Then, the K-means++ algorithm is used to cluster the pixels in the image.

The sizes of different regions in the image are different, and the eye is generally more attentive to large regions, ignoring the smaller ones. In accord with this property, the model considers the contribution of the size of each region to the region's saliency, and sets a size weighting for *R*_*k*_:

$$p_{k}=n_{k}/N$$ (3)

In the formula, *n*_*k*_ indicates the number of pixels in the region *R*_*k*_, and *N* represents the number of pixels in the *W* × *H* image.

### 2.1 Analysis of region saliency

Image contrast is the relative change in the value of adjacent pixels: the greater the change, the higher the contrast. Contrast is a key factor affecting the image's visual effect: generally, an image with higher contrast is clear and bright; otherwise, the image is blurred. As the eye is more sensitive to areas of high contrast, a region's pixel saliency can be estimated through an analysis of the region's contrast. The region is represented by *μ*, which is the mean value of the region's pixels in the feature space, and the contrast of region *R*_*k*_ is defined by incorporating the eye's attention response to the area:

$$\omega_{c}\left(k\right)=\sum_{i=1,i\neq k}^{K}\left(p_{k}{\left\|\mu_{k}-\mu_{i}\right\|}_{2}\right)$$ (4)

In the proposed model, the contrast *ω*_*c*_(*k*) of region *R*_*k*_ is weighted with respect to all regions of the image; it is an extension of the local contrast, which belongs to the overall contrast. The magnitude of the value shows the extent to which the region attracts more of the eye's attention than other regions; higher values indicate that the region is relatively more important for image analysis and understanding from a pixel perspective.

For the human visual system, the area near the center of an image attracts more attention, and the image boundary region is often neglected. As the distance between the object and the image center increases, the attention paid to the object decreases. This is called the *center deviation rule* of the image saliency detection algorithm. In our model, according to the above principle for the human eye, the spatial saliency for region *R*_*k*_ is represented by *ω*_*s*_(*k*), which is defined as:

$$\omega_{s}\left(k\right)=\frac{1}{n_{k}}\sum_{z_{i}\in R_{k},i=1}^{n_{k}}\exp\left({\frac{\left(z_{i}-o\right)}{2\sigma^{2}}}^{2}\right)$$ (5)

Therein, *o* represents the center of the image, and the variance *σ*^2^ is the normalized radius of the image. *z*_*i*_ indicates the *i*th pixel in the region *R*_*k*_. According to the 3*σ* principle, *σ* is determined by the size of the image:

$$\sigma =\frac{1}{6}\max\left(W,H\right)$$ (6)

### 2.2 Mapping of salient regions

The image region contrast *ω*_*c*_(*k*) and spatial saliency *ω*_*s*_(*k*) respectively reflect the contribution of the region's pixels and that of the relative spatial position to the human eye. The eye often combines the above two features to determine the importance of a region. Common ways of combining contrast and spatial saliency are linear summation or point by point multiplication. Point by point multiplication is better than linear summation for suppressing the noise, but linear summation can obtain a higher recall rate. For the saliency test, the accuracy rate is more important than the recall rate. In our model, the probability *p*(*k*) of joint saliency for region *R*_*k*_ is obtained by multiplying the integration contrast and the spatial saliency:

$$p(k)=\omega_{c}(k)\omega_{s}(k)$$ (7)

The regional contrast *ω*_*c*_(*k*) of our model is based on the calculation of the mean feature space of the region, without considering the contrast for each pixel in the region. Whereas the regional spatial saliency *ω*_*s*_(*k*) represents the weighted saliency of each pixel in the region relative to the image center, the joint salient probability *p*(*k*) only indicates each region's saliency, without indicating the regional saliency of each pixel. In our model, it is assumed that the pixels in the region are subject to the Gaussian distribution, and the saliency of pixel *z* in region *R*_*k*_ is

$$p\left(z|k\right)=\exp\left(\frac{{\left(z-\mu_{k}\right)}^{2}}{2\sigma_{k}^{2}}\right),\;z\in R_{k}$$ (8)

Therein, the variance $\sigma_{k}^{2}$ is the pixel variance of the region *R*_*k*_. The pixel saliency probability *p*(*z*) is obtained as the sum of all the regions jointly with saliency *p*(*z*|*k*)*p*(*k*):

$$p\left(z\right)=\sum_{k=1}^{m}p\left(z|k\right)p\left(k\right)$$ (9)

**Fig 1:**
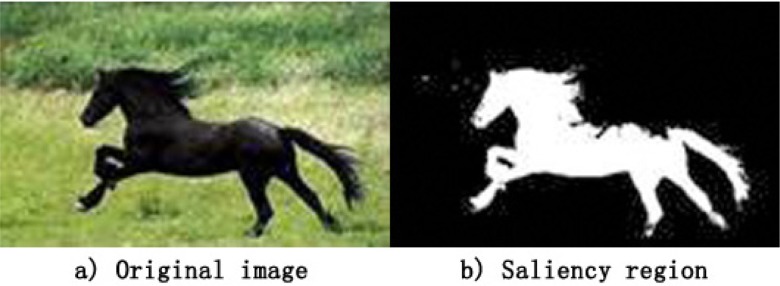
The salient region.

The image saliency detection results are shown in Fig 1. Fig 1B shows that the saliency using regional pixels and relative position estimation indicates only the importance of regional information and does not indicate whether the area has clear semantic meaning; however, the contour lines of salient regions are adjacent to the local edge lines of the object.

## Segmentation of salient objects

In order to segment the semantic object from the image, this analysis assumes that there is no dividing line between objects. As for local neighborhood, the object boundary is represented as the margin of local neighborhood. Meanwhile, in the process of image segmentation, by the deformation of the topological structure of the segmentation curve based on the object contour edges, the salient objects of the image are segmented by the active contour segmentation algorithm. The object segmentation of this algorithm is implemented by curve evolution. In this model, *φ*: Ω→*R* represents the Lipschitz function of three-dimensional space; for its level set representation curve *C*: *φ*(*x*,*y*) < 0 indicates an object within the region, and *φ*(*x*,*y*) > 0 indicates an external object. Its essence is to use function symbols to represent different regions. In order to facilitate the calculation, the Heaviside function *H*(*φ*) is introduced to represent the inner and outer regions of the curve, and the curve *C* can be expressed as *δ*(*φ*), which is the measure of Dirac, and the Dirac is the derivative of *H*(*φ*). *H*(*φ*) and *δ*(*φ*) are respectively given as follows:

$$H\left(\varphi \right)=\left\{ \begin{array}{ll} 1 & \varphi \geq 0\\ 0 & \varphi <0 \end{array}\right.\;,\;\delta (\varphi )=\frac{dH(\varphi )}{d\varphi }$$ (10)

According to the local marginal of the object contour, the edge curves are seen as constraint conditions, and the segmented curve evolutionary energy function of image Ω→[0,1] is

$$\varepsilon \left(\varphi \right)=\underset{\varphi }{\arg\;\min}\left\{\lambda \int_{\Omega }g\delta \left(\varphi \right)\left|\nabla \varphi \right|dxdy+\nu \int_{\Omega }gH\left(-\varphi \right)dxdy+\frac{\mu }{2}{\int_{\Omega }\left|\nabla \varphi -1\right|}^{2}dxdy\right\}$$ (11)

The first term in the formula represents the curve length; the second term represents the object region area, and the third term is the curve regularization term. *g* is the image edge indication function:

$$g={\left(1+\left|\nabla G_{\sigma }*u\right|\right)}^{-1}$$ (12)

If the curve is located in the smooth region, the gradient amplitude approaches zero, the edge indicator function tends to 1, and *ε*(*φ*) is larger; if the curve is located at the edge, the gradient is larger, *ε*(*φ*) is at a minimum, and the curve stops evolving.

In the traditional active contour algorithm, the initial curve is usually given first. If the initial curve is far from the object contour, the curve evolution time is longer. In our model, the region having a pixel saliency probability of more than 60% is seen as the initial region, and according to the region, the initial level-set function is defined as:

$$\varphi_{0}\left(z\right)=\left\{ \begin{array}{ll} -4 & \;p(z)\geq \max\left\{p(z)\right\}\\ 4 & \;\mathrm{otherwise} \end{array}\right.$$ (13)

In this study, the fast descent algorithm was used for the discrete calculation (formula 12), and the negative gradient of *φ* was calculated by the forward difference method. The discrete calculation of ∂*φ*/∂*t* is

$$\frac{\varphi_{i,j}^{k+1}-\varphi_{i,j}^{k}}{t}=\mu \left[\Delta \varphi_{i,j}^{k}-div\left(\frac{\nabla \varphi_{i,j}^{k}}{\left|\nabla \varphi_{i,j}^{k}\right|}\right)\right]+\lambda \delta_{a}\left(\varphi_{i,j}^{k}\right)div\left(g_{i,j}\frac{\nabla \varphi_{i,j}^{k}}{\left|\nabla \varphi_{i,j}^{k}\right|}\right)+\nu g_{i,j}\delta_{a}\left(\varphi_{i,j}^{k}\right)$$ (14)

In order to avoid this situation, this paper defines a confidence level Pr for the segmented regions according to the different segmentation regions with different smoothing components:

$$\mathrm{Pr}=\frac{card\left(A_{k}\cap A_{k-1}\right)}{\max\left\{card\left(A_{k}\right),card\left(A_{k-1}\right)\right\}}$$ (15)

Therein, *A* indicates the segmentation curve and its internal region {(*x*,*y*)|*φ*(*x*,*y*) ≤ 0}. When the confidence for the segmented region meets the following condition, it indicates that the desired degree of similarity between the two iterative segmentation results is achieved, and thus the smoothing iteration will be stopped:

Pr≥T (16)

*T* is the segmentation region confidence threshold, and its value is close to 1. The segmentation results are different for different confidence thresholds.

## Experiment, results, and analysis

The hardware environment of the experiment was an Intel Core^TM^ i3-3220 processor with 4GB memory. The operating system was Microsoft Windows 7, and the experimental simulation environment was Microsoft Visual C++ 6.0. In this model, the image is divided into different regions via a clustering algorithm, and the number of cluster regions *m* needs to be given in advance, but initially the number of salient objects in different images is not known. *m* is selected by the method of this paper. For this study, 2000 natural images from the Internet were segmented using different *m* values, and Table 1 shows the F-measures obtained from different settings of *m* values. The F-measures are presented in the form of ‘average±standard deviation’, and the experimental results in Table 1 reveal that the best average F-measure is achieved under the setting of *m* = 6.

**Table 1:** Average F-measures obtained from different settings of *m*.

	*m* = 3	*m* = 4	*m* = 5	*m* = 6	*m* = 7	*m* = 8	*m* = 9
F-measure	0.9494±0.019	0.9464±0.018	0.9477±0.02	0.9650±0.022	0.9521±0.017	0.9496±0.017	0.9407±0.018

**Fig 2:**
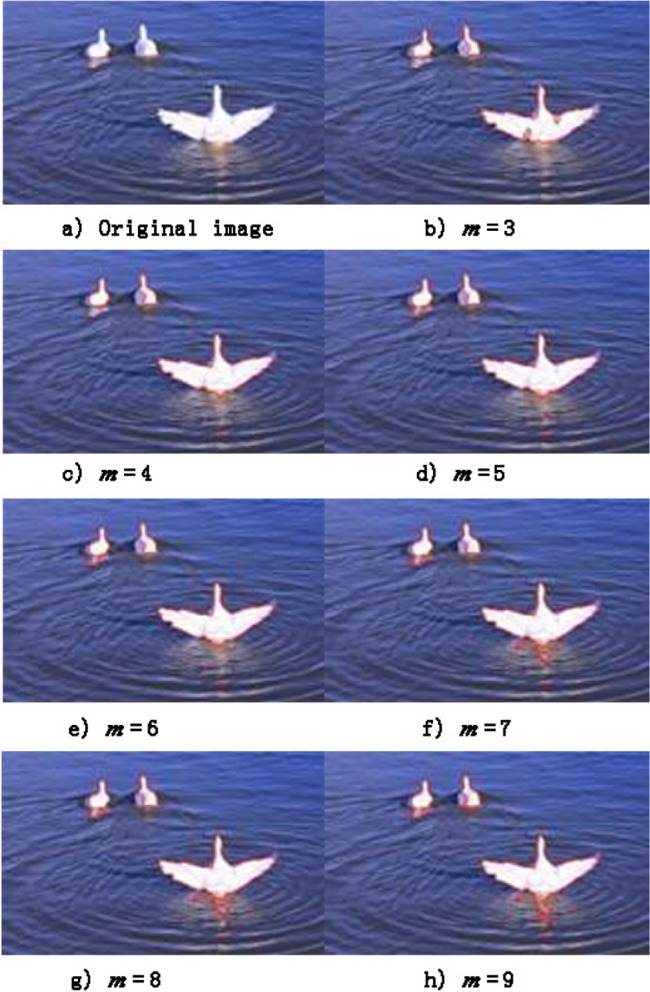
Results of segmentation using different values of *m* (the number of cluster regions).

To better illustrate the segmentation effects under different settings of *m*, Fig 2 shows the results of the segmentation of an image using different *m* values, and the segmentation effect is best when *m* = 6. The original image contains 3 visually salient objects, a semi-transparent region of 2 salient objects, and the background region containing the texture. If *m* = 3, the semi-transparent region is mis-segmented into background; if *m* = 9, the texture region is segmented as an object. (Fig 2E). If *m* = 6, the proposed algorithm will segment the semi-transparent regions of Fig 2A into salient objects.

In order to validate the effectiveness of the segmentation algorithm, the F-measures obtained by the proposed algorithm are compared with those of the method proposed in reference [19]. In order to check if the proposed algorithm is significantly better than the method given in reference [19], the Wilcoxon paired signed-rank test is conducted to compare the performance of two methods. The test results are reported in Table 2. The Wilcoxon test rejects the hypothesis of equivalence with significance level at *p* = 0.000, indicating our proposed method is significantly better than the method given in reference [19].

**Table 2:** Wilcoxon pairwise test result of our method vs. method in reference [19].

	Our method	Method in (19)	*p-value*
Avg F-measure	0.9650	0.9324	*p* = 0.000<0.05

**Fig 3:**
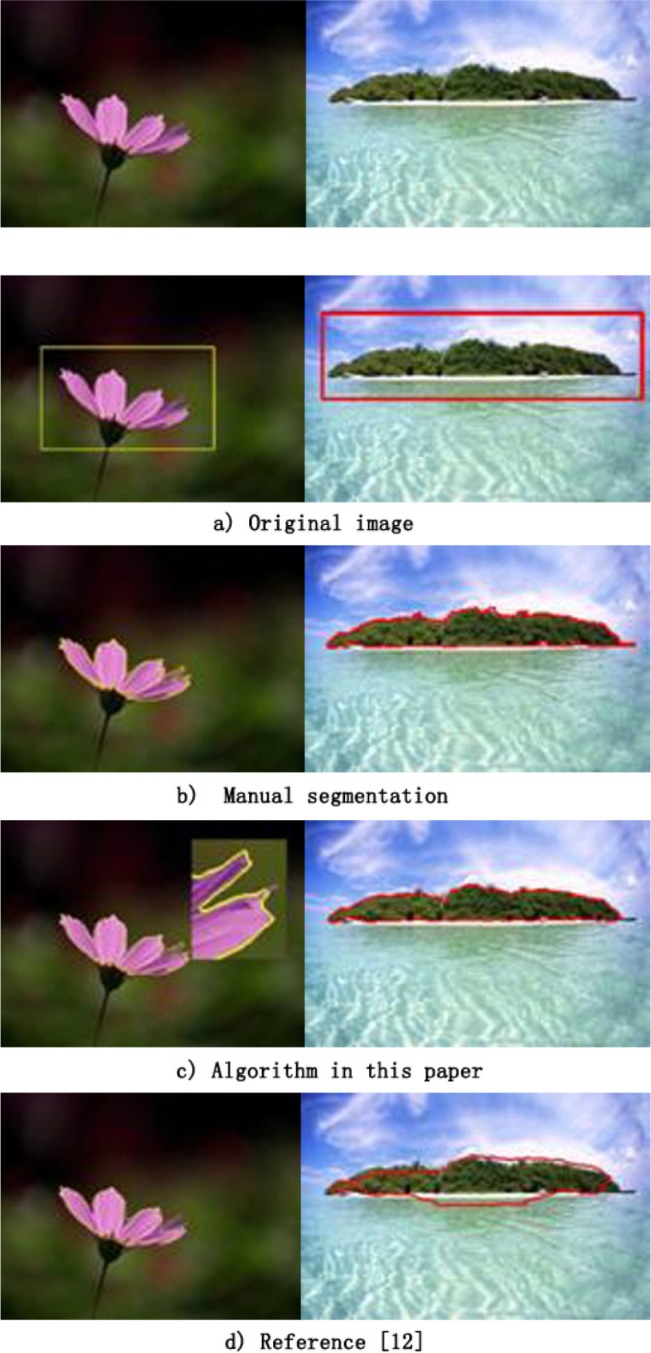
Results of segmentation on images with a single object.

To better illustrate the performance of our method, we show the segmentation results on images with different number of salient objects. We run our method and the method given in in reference [19] on an image with one salient object, and Fig 3 shows the results. In Fig 3, there is only one salient object. The brightness of the background region of the left image is nearly constant, and there is a change in the brightness of the background region of the right image. The initial curve (adopting the algorithm of reference [19]) is in the original image (Fig 3A). Fig 3B shows the manual segmentation results. Fig 3C indicates the results using the algorithm proposed in this paper. Here, the left (800×600) image segmentation F-measure is 0.976, and the system time was 2.36 s. In addition to the cusp, the segmentation curve is accurate, and the curve of the cusp is over-convergent because the active contour segmentation algorithm requires that the curve be smooth. For the right (320×600) image, the segmentation F-measure is 0.987, and the system time was 1.89 s. Fig 3D shows the segmentation results using the method of reference [19]. Here, the F-measures of the left and right side image segmentations are 0.889 and 0.825, respectively, and the system operation times were 16.36 and 14.89 s, respectively. With the proposed method, the salient region boundary is adjacent to the contour of the target, and the curve evolution time was shorter.

**Fig 4:**
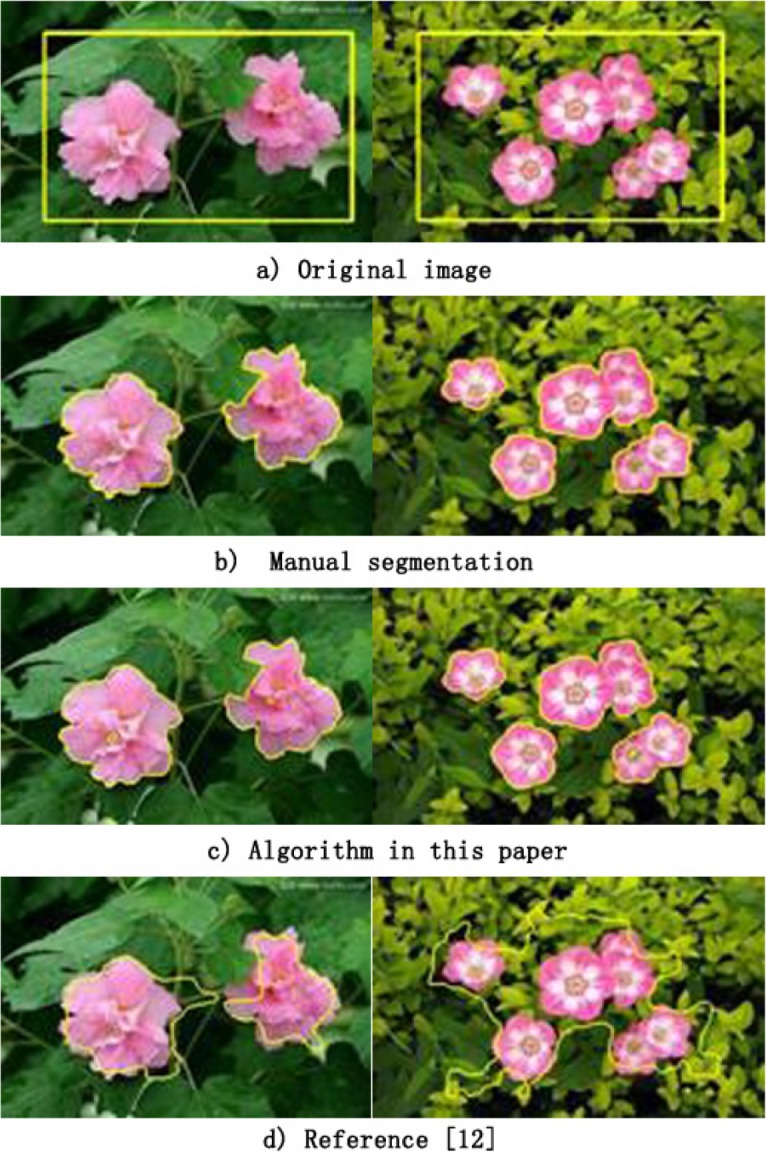
Results of segmentation on images with complex backgrounds.

In order to validate the effectiveness of the proposed algorithm for the segmentation of images with complex backgrounds, the images shown in Fig 4 were segmented, and the results are compared with those of the method given in reference [19]. In both images of Fig 4, there are at least 2 salient objects, the background is complex, and the initial curve (adopting the algorithm of reference [19]) is in the original image (Fig 4A). Fig 4B shows the manual segmentation results. Fig 4C shows the results using the proposed algorithm; the left (344×233) image segmentation F-measure is 0.986, and the system time was 1.36 s. The right (360×227) image segmentation F-measure is 0.992, and the system time was 1.89 s. Fig 3D shows the results of the segmentation method given in reference [19]; the left and right side image segmentation F-measures are 0.859 and 0.676, respectively, and the system times were 6.36 and 4.89 s, respectively. From the perspective of system operation time, the curve evolution time is shorter using the proposed method.

## Conclusion

By an organic combination of region saliency analysis with the active contour segmentation algorithm, the salient object segmentation model is established in this paper. Using a priori background knowledge, the pixels are clustered for each region by the K-means++ algorithm; the attention mechanism of the human eye is used to analyze regional contrast and spatial saliency probabilities and to highlight the saliency of the segmentation regions, and the level-set algorithm is used to perform salient object segmentation of the image. With this method, each region segmented by our algorithm has clear semantic meaning, and its importance is emphasized; in addition, the salient region boundary is adjacent to the object contour, so the curve evolution time is shorter. However, when the image pixels are divided into different regions, the edge information of the image is not considered.
